# A simple score to estimate the likelihood of pseudoprogression vs. recurrence following stereotactic radiosurgery for brain metastases: The Bergen Criteria

**DOI:** 10.1093/noajnl/vdaa026

**Published:** 2020-03-10

**Authors:** Bente Sandvei Skeie, Per Øyvind Enger, Jonathan Knisely, Paal-Henning Pedersen, Jan Ingeman Heggdal, Geir Egil Eide, Geir Olve Skeie

**Affiliations:** 1 Department of Neurosurgery, Haukeland University Hospital, Bergen, Norway; 2 Department of Neurosurgery, Stavanger University Hospital, Stavanger, Norway; 3 Department of Radiation Oncology, Weill Cornell Medical College, New York, NY, USA; 4 Department of Oncology and Medical Physics, Haukeland University Hospital, Bergen, Norway; 5 Department of Global Public Health and Primary Care, University of Bergen, Norway; 6 Centre for Clinical Research, Haukeland University Hospital, Bergen, Norway; 7 Department of Neurology, Haukeland University Hospital, Bergen, Norway

**Keywords:** brain metastases, pseudoprogression, radiation necrosis, stereotactic radiosurgery, tumor recurrence

## Abstract

**Background:**

A major challenge in the follow-up of patients treated with stereotactic radiosurgery (SRS) for brain metastases (BM) is to distinguish pseudoprogression (PP) from tumor recurrence (TR). The aim of the study was to develop a clinical risk assessment score.

**Methods:**

Follow-up images of 87 of 97 consecutive patients treated with SRS for 348 BM were analyzed. Of these, 100 (28.7%) BM in 48 (53.9%) patients responded with either TR (*n* = 53, 15%) or PP (*n* = 47, 14%). Differences between the 2 groups were analyzed and used to develop a risk assessment score (the Bergen Criteria).

**Results:**

Factors associated with a higher incidence of PP vs. TR were as follows: prior radiation with whole brain radiotherapy or SRS (*P* = .001), target cover ratio ≥98% (*P* = .048), BM volume ≤2 cm^3^ (*P* = .054), and primary lung cancer vs. other cancer types (*P* = .084). Based on the presence (0) or absence (1) of these 5 characteristics, the Bergen Criteria was established. A total score <2 points was associated with 100% PP, 2 points with 57% PP and 43% TR, 3 points with 57% TR and 43% PP, whereas >3 points were associated with 84% TR and 16% PP, *P* < .001.

**Conclusion:**

Based on 5 characteristics at the time of SRS the Bergen Criteria could robustly differentiate between PP vs. TR following SRS. The score is user-friendly and provides a useful tool to guide the decision making whether to retreat or observe at appropriate follow-up intervals.

**Key Points**

- Treatment data predict the risk of pseudoprogression vs. recurrence post-SRS.
- The Bergen Criteria robustly assesses the risk of pseudoprogression vs. recurrence.

**Importance of the Study**

A key task in the follow-up of patients with brain metastases (BM) treated with stereotactic radiosurgery (SRS) is to differentiate pseudoprogression (PP) from recurrence, for which treatment and prognosis are different. Nearly 1/3 of BM treated with SRS will display increased contrast enhancement on standard magnetic resonance imaging; either temporarily due to successful SRS or progressively due to failed SRS. Previous studies have focused on sophisticated imaging techniques to distinguish radiation induced changes from recurring tumors. The present study is the first to evaluate clinical data to predict the risk of developing PP vs. recurrence. Known predictors for successful SRS (small volume, radiosensitive histology, prior radiation, and optimal dosimetry) intuitively predict higher risk of PP vs. recurrence. Based on the presence or absence of these 4 predictors the Bergen Criteria was established. It estimates with high accuracy the likelihood of PP vs. recurrence, is user-friendly and cost-free. Further validation of the Bergen Criteria is warranted.

A major challenge in the management of patients with brain metastases (BM) treated with stereotactic radiosurgery (SRS) is to distinguish radiation necrosis or pseudoprogression (PP) from tumor recurrence (TR) for which treatment and prognosis are different^1,2^ (Figure 1A). TR is due to failed SRS and further treatment is needed. PP on the other hand is a sign of successful SRS due to radiation induced damage to the blood–brain barrier^3^ and influx of inflammatory cells.^4,5^ These changes lead to increased T1-contrast enhancement on magnetic resonance imaging (MRI) mimicking TR, but subside spontaneously without change in treatment.^6^ Ambiguous MRI changes during follow-up are often managed by conducting an additional, early MRI follow-up to see if the changes stabilize or subside^7,8^ and/or to add additional imaging techniques^9^ to try to differentiate between the two. MRI perfusion^10,11^ may show high intratumoral blood perfusion in TR and Positron Emission Tomography (PET)^12–14^ may show increased uptake of glucose or amino acids in TR compared with PP, respectively, but differentiation can still be difficult. In some cases a biopsy^15^ or resection of the lesion will provide a definite diagnosis but involves the risks associated with a surgical procedure. Moreover, a wait and see strategy may lead to delayed treatment in cases with TR, whereas immediate treatment may turn out to be unnecessary if PP and even harmful if the lesion is reirradiated.^16^

**Figure 1:**
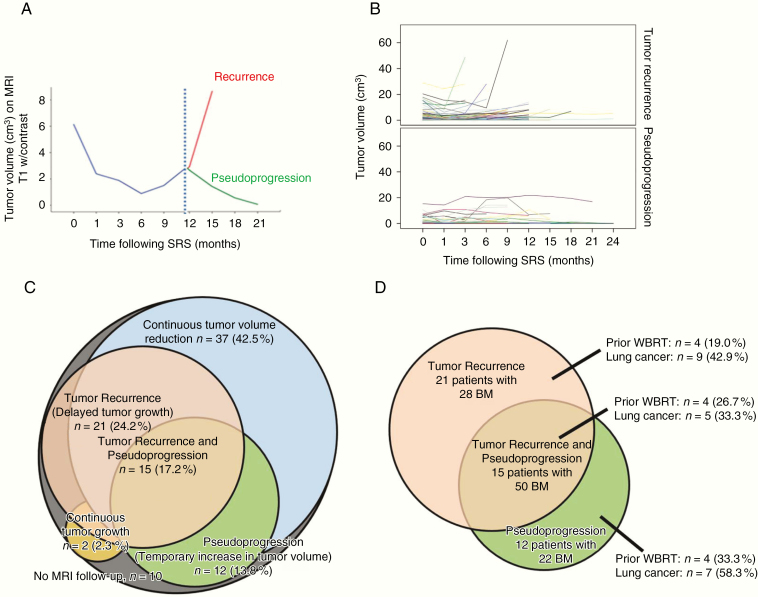
(A) Illustration of 2 potential response patterns for brain metastases (BM) treated with stereotactic radiosurgery (SRS): pseudoprogression (PP) and tumor recurrence (TR). (B) Individual tumor volume response curves on contrast enhanced T1-weighted MRI at stereotactic radiosurgery (time: 0) and during follow-up for (A) 53 pseudoprogressing tumors and (B) 47 recurring tumors. (C) Venn diagram of 87 out of 97 patients with follow-up MRI of a total of 348 BM post-radiosurgery. The diagram illustrates the proportion of patients with BM responding with the 4 distinct volumetric response patterns on contrast enhanced MRI: tumor recurrence (red), pseudoprogression (green), continuous tumor volume reduction (blue), and continuous tumor growth (orange). (D) Venn diagram of patients (*n* = 48) included in the development of the Bergen Criteria. Tumor type (lung cancer vs. other primary sites) and prior irradiation history for patients in 3 cohorts are illustrated: 21 (43.8%) patients with 28 recurrences (red), 12 (25.0%) patients with 22 pseudoprogressions (green), and 15 (31.2%) patients with some recurrences (*n* = 25 BM) and some pseudoprogressions (*n* = 25 BM) following initial and/or repeat-SRS. MRI, magnetic resonance imaging.

It is well documented that the chance of successful SRS is higher if the BM are small,^17,18^ radiosensitive,^19^ previously irradiated^20^ and treated with optimal dosimetry.^17,21^ Thus, we wanted to investigate whether baseline tumor- and treatment-related data that are readily available in a clinical setting could predict the likelihood of PP vs. TR (when follow-up MRI shows increased contrast enhancement) and whether these data could be integrated into a clinical risk assessment score. To achieve this, we investigated the predictive value of various baseline characteristics at SRS to assess the likelihood of PP vs. TR.

## Methods

Volumetric tumor change was a secondary end point in a prospective study evaluating quality of life changes following Gamma Knife Surgery (GKS) for BM.^22^ Treatment and follow-up MRI of 97 consecutive patients treated for a total of 406 BM with Gamma Knife Perfexion at Haukeland University Hospital between 2009 and 2011 were prospectively collected and analyzed. Two hundred and twenty-five BM were treated initially (iSRS), 160 BM at subsequent GKS (sSRS) as distant failures, and 21 BM were treated with repeat-SRS due local failure (LF) following iSRS. In the present study, we included all BM that either pseudoprogressed (47 BM) or recurred (53 BM), Figure 1B.

Ethical approval was obtained from the Regional Ethical Committee for Medical Research (REK, 2010/801) and written informed consent was obtained from all patients.

### SRS Treatment: Radiation Doses Used for Various Sizes of Metastases

BM were treated with a prescribed dose of 20–25 Gy (*n* = 340), and 16–18 Gy if prior radiation, large total tumor volume (>10–15 cm^3^) and/or close proximity to critical structures (*n* = 50). For 16 (4%) BM the prescription dose was ≤15 Gy due to prior SRS (*n* = 9), prior whole brain radiotherapy (WBRT) (*n* = 3), large tumor volume (*n* = 2), or tumor location (*n* = 2).

### Follow-up Schedule and Volume Measurements of SRS Treated BM

The BM volumes on MRI T1-contrast enhanced imaging (MRI-T1-C) were measured utilizing the GammaPlan software (Elekta, Stockholm, Sweden) at GKS and during follow-up at 1 and 3 months, then every third month post-GKS until September 30, 2019 or death. Follow-up images were available for 348 (85.7%) out of 406 BM in 87 (89.7%) of the 97 patients (95.6% of the 91 patients alive at first MRI 1 month post-SRS). Two patients (2.1%) are still alive in September 2019.

### Volumetric Tumor Response—Definition of PP vs. TR

The volume changes over time on MRI-T1-C from SRS until LF leading to new treatment (surgery, WBRT, or re-SRS) or until last follow-up/death was used to define PP from TR. Local failures that were retreated with a second course of SRS were evaluated as separate lesions/treatments and volume changes from re-SRS were recorded.

All BM followed 1 of 4 principal response patterns: either continuous reduction in size (*n* = 238, 68%), transient increase in volume defined as PP (*n* = 47, 14%), delayed growth defined as TR (*n* = 53, 15%), or no response with continuous growth (*n* = 10, 3%) (B.S.S. et al., unpublished manuscript). Only 2 (2%) of the BM were resected after SRS yielding biopsy material. Figure 1C illustrates the distribution of the different volumetric response patterns for all 87 patients (348 BM) with follow-up MRI.

### Patient Population Used to Develop a Risk Assessment Score for the Likelihood of PP vs. TR

We included all 48 (55.1%) out of the 87 patients with follow-up images of at least 1 BM that either pseudoprogressed (47 BM) and/or recurred (53 BM), Figure 1D. Thus, 100 (28.7%) out of 348 BM were included of which 86 were treated with iSRS and 14 of these with subsequent re-SRS due to TR. The mean age of the 48 included patients was 60 years (range 39–86 years) and 22 (45.8%) were males.

### Tumor Subtypes

The primary cancer site was lung (*n* = 21 patients [43.8%]), melanoma (*n* = 7 [14.6%]), renal (*n* = 5 [10.4%]), breast (*n* = 4 [8.3%]), colorectal cancer (CRC, *n* = 9 [18.8%]), or unknown (*n* = 2 [4.2%]).

### Prior Whole Brain Radiotherapy and Immunotherapy

Twelve (25%) of the 48 patients that had previously been irradiated with WBRT. Thirty of the included BM (30%) occurred in the 12 patients with prior WBRT. None of the patients in this cohort were treated with immune checkpoint inhibitors at the time of SRS.

### Repeat-SRS

Eleven (22.9%) of the 48 patients underwent iSRS (*n* = 14 BM) and later re-SRS (*n* = 14 BM) for the same BM due to recurrence. Post-re-SRS 12 of the 14 BM subsequently pseudoprogressed and 2 re-recurred. The remaining 37 (77.1%) patients only underwent iSRS for 72 BM; 12 patients had at least 1 BM that pseudoprogressed, 20 patients had at least 1 BM that recurred, and 5 patients had a mixture of PP and TR.

### The Bergen Criteria

All 47 BM responding with PP were compared with the 53 BM responding with TR. Baseline tumor- and treatment-related characteristics found to be associated with development of PP compared with TR were used to design a risk assessment score to estimate the likelihood of PP vs. TR (the Bergen Criteria). The score was refined by choosing predictive factor splits for maximizing practical use.

### Statistics

To analyze differences between BM responding with PP vs. recurring BM we used Student’s *t*-test for continuous variables (baseline BM volume, prescription dose, maximum dose, target cover ratio, 12 Gy normal brain volume, total steroid dose, and time from SRS until increase in BM volume on MRI-T1-C) and Pearson’s χ ^2^ test for categorical variables (primary cancer site, prior treatment with SRS and/or WBRT, and steroid medication). For variables found to be significantly associated with PP vs. TR we used Pearson’s χ ^2^ test to refine the score: prior SRS (yes or no), prior WBRT (yes or no), BM volume ≤2 cm^3^ (yes or no), target cover ratio ≥98% (yes or no), primary lung cancer (yes or no), and to assess the predictive value of the Bergen Criteria. Overall survival (OS) was analyzed by the Kaplan–Meier method. Date for end-of-follow-up was September 30, 2019.

## Results

The BM in the 4 response groups described above differed significantly in volume, prescribed dose, and target cover ratio at SRS (Table 1). There was a striking similarity between BM with favorable outcomes (continuous tumor volume reduction or PP) and BM with unfavorable outcomes (continuous tumor growth or TR). BM with a favorable outcome were generally small (mean volume ≤2.3 cm^3^) and treated with high target cover ratio (mean target cover ratio ≥ 98%) at SRS. On the other hand, BM with an unfavorable outcome were large (mean volume ≥3.5 cm^3^) and less optimally covered at SRS (mean target cover ratio <98%). We found significant differences when comparing BM in the PP group with the TR group: First, they were significantly smaller (mean BM volume 2.3 vs. 4.8 cm^3^, *P* = .015) and more completely covered with the prescribed dose (mean target cover ratio 98.6% vs. 97.6%, *P* = .026). These differences at baseline were used to predict the likelihood that increased MRI-T1-C enhancement following SRS was due to PP or recurrence.

**Table 1:** Baseline Characteristics for Brain Metastases (BM) According to 4 Principle Tumor Volume Response Curves on Contrast Enhanced T1-Weighted-MRI Following Stereotactic Radiosurgery (SRS) in 348 of 406 BM With Follow-up MRI (in 87 of 97 Consecutive Patients) at Haukeland University Hospital in Bergen (Norway) Between 2009 and 2011

Characteristic	Response Curve Group	*n* (%)	Mean	*P* Value
Tumor volume at SRS (cm^3^)	Continuous tumor volume reduction	238 (68)	1.4	<.001
	Temporary increase in tumor volume (PP)	53 (15)	2.3	
	Delayed growth (TR)	47 (14)	4.8	
	Continuous tumor growth	10 (3)	3.5	
Prescription dose at SRS (Gy)	Continuous tumor volume reduction	238 (68)	20.5	<.001
	Temporary increase in tumor volume (PP)	53 (15)	20.2	
	Delayed growth (TR)	47 (14)	19.3	
	Continuous tumor growth	10 (3)	15.9	
Target cover ratio at SRS (%)	Continuous tumor volume reduction	238 (68)	98.9	<.001
	Temporary increase in tumor volume (PP)	53 (15)	98.6	
	Delayed growth (TR)	47 (14)	97.6	
	Continuous tumor growth	10 (3)	96.0	

MRI, magnetic resonance imaging; PP, pseudo-progression; TR, tumor recurrence.

### Baseline Tumor Volume as Predictor for PP vs. TR

**Figure 2:**
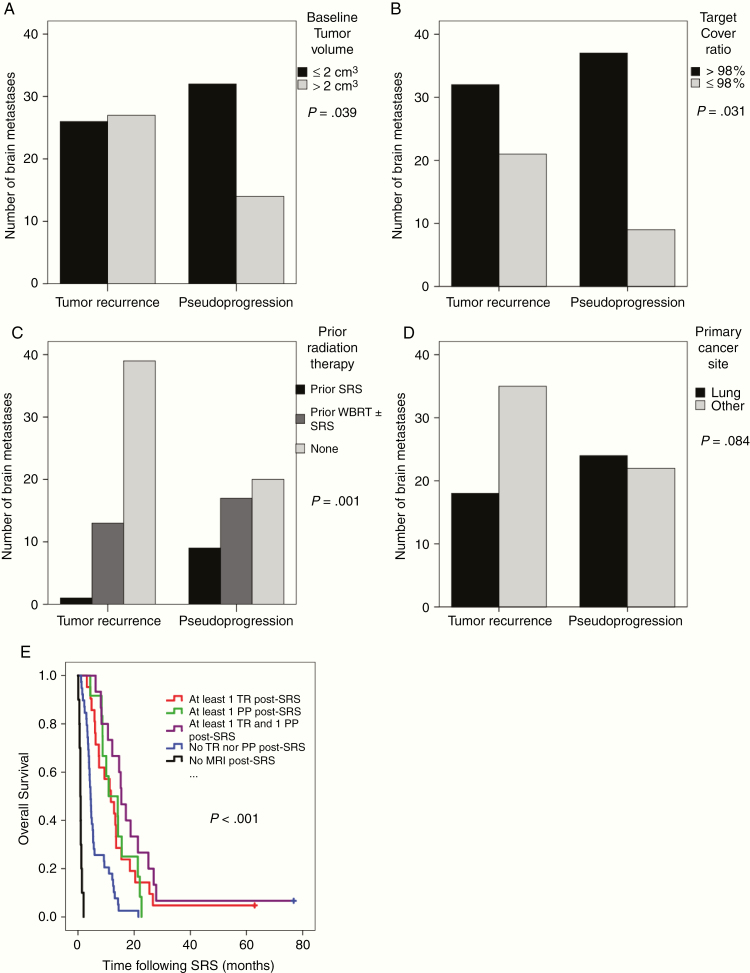
The number of brain metastases (BM) that responded to stereotactic radiosurgery (SRS) with pseudoprogression or tumor recurrence with: (A) Baseline volume ≤2 cm^3^ vs. >2 cm^3^. (B) Target cover ratio at SRS <98% vs. ≥98%. (C) Prior treatment with SRS, whole brain radiotherapy ± SRS vs. no prior radiation treatment. (D) Primary lung cancer origin vs. other origin than lung cancer. (E) Overall survival curves for patients with (a) at least 1 BM that recurred (TR) (red), (b) at least 1 BM that pseudoprogressed (PP) (green), (c) a mixture of TR and PP (violet), (d) no TR nor PP (blue), and (e) no follow-up images (black).

For practical reasons we used 2 cm^3^ as cutoff. It corresponds to a tumor diameter of 1.5 cm that can be easily assessed on MRI. We found that BM less than 2 cm^3^ were more likely to develop PP than larger BM. Of the 47 BM displaying PP, 32 (68.1%) vs. 15 (31.9%) were ≤2 cm^3^, or less than 1.5 cm in diameter vs. >2 cm^3^ (*P* = .054, χ ^2^ = 3.7, df = 1, and Cramer’s *V*: 0.192). Similar numbers for recurring BM were 26 (49.1%) vs. 27 (50.9%), Figure 2A.

### Target Cover Ratio at SRS as Predictor for PP vs. TR

BM responding with PP had significantly more often a higher coverage with the prescribed dose at SRS compared with recurring BM. Using 98% as cutoff we found a significantly higher incidence of PP vs. recurrence if the target cover ratio was ≥98%. Of 46 BM displaying PP, 37 (78.7%) vs. 10 (21.3%) had a target cover ratio of ≥98% vs. <98% and for recurring BM 32 (60.4%) vs. 21 (39.6%), *P* = .048, χ ^2^ = 3.9, df = 1, and Cramer’s *V*: 0.198, Figure 2B.

### Prior Radiation Treatment as Predictor for PP vs. TR

Prior IR was a strong predictor of PP vs. TR (*P* = .001, χ ^2^ = 13.7, df = 2, and Cramer’s *V*: 0.370). In the PP group, 10 BM (21.3%) were previously treated with SRS, 17 (36.2%) with WBRT whereas 20 (42.6%) were not previously irradiated. On the contrary, in the TR group, only 1 BM (1.9%) was previously treated with SRS, 13 (24.5%) with WBRT, and 39 (73.6%) were not previously irradiated, Figure 2C.

### Primary Cancer Site as Predictor for PP vs. TR

We found that BM in the PP group more often originated from primary lung cancer than from other primaries compared with BM in the TR group (*P* = .084, χ ^2^ = 3.0, df = 1, and Cramer’s *V*: 0.173). The primary cancer site was lung for 24 (57.1%) of the 47 BM in the PP group and 18 (34.0%) of the 53 BM in the TR group, Figure 2D.

### Time Frame for Detection of PP vs. TR

The mean time from SRS until increased contrast enhancement on MRI was detected was significantly shorter for PP than TR, 5.1 months (SD 4.1, range 1–18 months) vs. 8.4 months (SD 6.5, range 3–36 months), respectively, *P* = .003. The mean time for the contrast enhancement to subside in PP was 3.2 months (SD 1.4, range 1–6 months). PP occurred within 6 months of SRS for 37 (78.7%) of 47 BM and more than 6 months post-SRS for 10 (21.3%). Similar numbers for TR are 31 (58.5%) and 22 (41.5%) of 53 BM, respectively (*P* = .030, χ ^2^ = 4.7, df = 1, and Cramer’s *V*: 0.216).

### Steroid Treatment as Predictor for PP vs. TR

Neither the use of steroids at SRS (yes vs. no; *P* = .897) nor the steroid dose used at baseline (categorical variables: 0 vs. 1–4 vs. >4 mg dexamethasone; *P* = .748/continuous variable: total steroid dose; *P* = .930) was significantly associated with development of PP (mean 4.0 mg, SD 6.0, range 0–16 mg) vs. TR (mean 5.0 mg, SD 5.2, range 0–16 mg).

### The Bergen Criteria for Assessing the Risk of PP vs. Recurrence

Factors associated with a higher incidence of PP vs. TR used were as follows (1) prior SRS, (2) prior WBRT, (3) target cover ratio ≥98%, (4) BM volume ≤2 cm^3^, and (5) primary lung cancer vs. other primaries. Based on the presence (0) or not (1) of these 5 parameters, the Bergen Criteria for risk assessment of PP vs. TR was established (Table 2). The total score ranges from 0 to 5 points. A score of 0 corresponds with high risk of PP vs. TR, whereas a score of 5 corresponds with a low risk of PP vs. TR. For simplicity, the predictive value of 4 Bergen Criteria groups, total score <2, 2, 3, and >3 points, respectively, was calculated (Table 3).

**Table 2:** The Pseudoprogression (PP) Risk Assessment Score (the Bergen Criteria), With Total Range: 0 (Low Risk of PP) to 5 (High Risk of PP), Is Defined as the Sum of the Scores on 5 Baseline Characteristics Based on 348 of 406 BM With Follow-up MRI (in 87 of 97 Consecutive Patients) at Haukeland University Hospital in Bergen (Norway) Between 2009 and 2011

Baseline Characteristics	Bergen Criteria Scores	
	0	1
Primary lung cancer	Yes	No
BM volume ≤2 cm^3^ (≤1.5 cm in diameter)	Yes	No
Target cover ratio >98%	Yes	No
Prior SRS to the same BM	Yes	No
Prior WBRT	Yes	No

BM, brain metastases; MRI, magnetic resonance imaging; SRS, stereotactic surgery; WBRT, whole brain radiotherapy.

**Table 3:** The Likelihood of Pseudoprogression (PP) vs. Tumor Recurrence (TR) as a Cause of Tumor Enlargement on Contrast Enhanced T1-Weighted-MRI Following Stereotactic Radiosurgery (SRS) (*n* = 100 Brain Metastases in 48 Patients) According to the Bergen Criteria at Haukeland University Hospital in Bergen (Norway) Between 2009 and 2011

The Bergen Criteria	Total BM With PP or TR (*N* = 100)	Likelihood of PP (%) (*n* = 47)		Likelihood of TR (%) (*n* = 53)	
	*N* (%)	%	*n* (% of PPs)	%	*n* (% of TRs)
0–1	12 (12.0)	100	12 (25.5)	0	0 (0.0)
2	28 (28.0)	57	16 (34.0)	43	12 (22.6)
3	35 (35.0)	43	15 (31.9)	57	20 (37.7)
4–5	25 (25.0)	16	4 (8.5)	84	21 (39.6)

BM, brain metastases; MRI, magnetic resonance imaging. The Bergen Criteria: pseudoprogression risk assessment score, ie, the sum of 5 baseline characteristics (Table 2).

A score of <2 point was associated with a 100% likelihood of PP, 2 points with 57% risk of PP vs. a 43% risk of TR, 3 points with a 57% risk of TR vs. 43% risk of PP whereas >3 points were associated with 84% likelihood for TR vs. 16% for PP (χ ^2^ = 24.6, df = 3, *P* < .001, and Cramer’s *V* = 0.496).

All BM with Bergen Criteria score >3 originating from primary CRC (*n* = 6), lung cancer (*n* = 3), or unknown primary (*n* = 2) belonged to the TR group vs. 71% (*n* = 10) of the 15 BM origination from other primaries (melanoma, renal, and breast cancer). All 7 BM with Bergen Criteria >3 and contrast enhancement occurring >6 months after SRS belonged to the TR group vs. 14 of the 18 BM occurring ≤6 months after SRS.

The Bergen Criteria is applicable also when excluding the 13 retreated BM. For the 87 BM that were not retreated Bergen Criteria 0–1 were associated with 100% PP and Bergen Criteria of 2, 3, and 4–5 with 52%, 42%, and 12% PP, respectively, *P* < .001).

The mean scores for the Bergen Criteria for the 4 response patterns, continuous reduction in size, PP, delayed growth (TR), and continuous growth, are 2.7 (SD: 0.72, range 1–4), 2.3 (SD: 0.95, range: 1–4), 3.2 (SD: 0.78, range 2–4), and 3.1 (SD: 0.74, range 2–4), respectively (χ ^2^: 20.5, df: 3, *P* < .001).

### Treatment at PP and TR Following SRS

None of the BM responding with PP were resected, 43 (91.5)% were asymptomatic, and 4 (8.5%) were managed with a short course of dexamethasone treatment.

Twenty-nine (52.7%) out of 53 TRs were treated conservatively with observation, 18 (32.7%) were retreated with SRS, 2 (3.6%) were resected with biopsy confirmed TR, and 4 (7.3%) were treated with WBRT.

### Overall Survival

The OS for patients with TR, PP, or both was significantly longer than for the 49 patients with MRI follow-up that experience neither TR nor PP, *P* < .001. The median OS for the last group was only 4.5 months (95% CI 3.8–5.1) compared with 11.7 months (95% CI 6.6–16.8) for the 21 TR only patients, 10.9 months (95% CI 3.9–17.9) for the 12 PP only patients, and 15.4 months (95% CI 12.4–18.5) for the mixed response group, respectively. The OS for the 10 patients without MRI follow-up was 0.83 months (95% CI 0.4–1.2), Figure 2E.

## Discussion

Differentiating TR from PP is a daily clinical challenge and of utmost importance to avoid both unnecessary treatments and treatment delays. More than 50% of the patients in our material experienced either TR or PP of at least one of their BM, which is in line with previous studies.^23^ One could argue that PP is due to successful or even “overtreatment” with SRS while TR reflects failed SRS. Utilizing treatment data is a novel way of distinguishing PP from TR. From our data we found 4 distinct baseline characteristics at SRS distinguishing BM responding with PP from recurring BM. They were more often previously irradiated, significantly better covered with the prescribed dose at SRS, smaller and more often originating from primary lung cancer. Using this information we were able to create a simple score which robustly distinguished PP and TR in a large number of patients. All BM in our study responding either with PP or TR with a score less than 2 belonged to the PP group. For the BM with a score more than 3, 84% belonged to the TR group. The Bergen Criteria is based on easily accessible tumor- and treatment-related characteristics available in the patient’s medical record and does not require special training or additional advanced imaging techniques. Furthermore, the Bergen Criteria is based purely on known predictors^2^ for successful SRS and is therefore intuitive. We know when we treat a large radioresistant brain metastasis with an incomplete target cover ratio that the chance of success is lower than if we treat a small, previously irradiated radiosensitive brain metastasis with optimal target cover ratio. The Bergen Criteria works as follows. If a brain metastasis ≤2 cm^3^ originating from lung cancer that was irradiated with WBRT prior to SRS starts to enlarge on MRI-T1-C, we can be 100% certain that this is due to PP if the brain metastasis was completely covered with the prescribed dose (Bergen Criteria score 0). Similarly, if a brain metastasis >2 cm^3^ at SRS originating from melanoma and not previously irradiated starts to enlarge following SRS, we can be 84% confident that the BM are recurring if the BM were not fully covered at SRS (Bergen Criteria score 5). The likelihood might further be estimated to 100% if the primary cancer is CRC, unknown or lung or the contrast enhancement is detected more than 6 months post-SRS, but this needs to be confirmed in a larger cohort of patients.

The Bergen Criteria can classify all BM enlarging following SRS either as most likely due to PP or most likely due to TR. Depending on the total score the risk of TR varies from 0% to 84% and the risk of PP from 16% to 100%. If TR is most likely it may be an advantage to confirm whether an enlarging tumor on MRI-T1-C follow-up is due to PP or TR with additional imaging such as the T1/T2 mismatch^8,24^ before initiating treatment. If the tumor size is comparable on contrast enhanced T1- and T2-weighted MRI images, this is most likely to represent the true tumor size and hence TR. If however, the BM are smaller on the T2- than T1-contrast series, the difference in size on the T1 vs. T2 is most likely due to changes in the normal brain–blood circulation surrounding the tumor and hence reflect PP. MRI perfusion and PET may be of value as reduced blood flow and uptake of glucose or amino acids strengthens the suspicion of PP vs. TR. Advanced imaging methods and biopsies evaluate the tumor at the time of progression or PP. However, the Bergen Criteria can estimate the chance of success based on treatment-related factors alone and thus adds value to the existing methods by combining information at the time of SRS with information at the time the BM volume starts to increase on MRI-T1-C post-SRS.

PP is considered a good prognostic sign.^23^ PP is often asymptomatic (>90% in our study) and symptoms usually respond to treatment with a short course of steroids. In rare cases, surgery may be necessary to relieve symptoms. Nevertheless, surgery has an inherent risk of complications, thus a wait and see strategy is preferred if one suspects PP.^2^ We suggest that the Bergen Criteria may be used to tailor appropriate follow-up intervals. If a BM enlarges on MRI-T1-C following SRS with a Bergen Criteria score <2, the patient may continue with standard 3 months follow-up intervals due to a high likelihood of PP. Conversely, a Bergen Criteria score >3 calls for an early MRI follow-up or immediate retreatment. If the score is 2 or 3, the patient may be followed at intermediate intervals (6–8 weeks) or the patient should be offered additional imaging with perfusion MRI or PET.

Radiation is the main cause of PP in BM patients. For a brain metastasis to achieve a Bergen Criteria score <2 it must have been previously irradiated either with SRS, WBRT, or both. There are few reports on PP following WBRT alone. PP seems to occur when WBRT is combined with SRS^25^ and when high-dose SRS is used alone or repeated for the same brain metastasis. Thus, the total accumulated dose to the tumor and normal brain is clearly related to the development of PP. In the present study, the likelihood of PP increased if the brain metastasis was completely covered with the prescribed dose independent of the dose itself. This implies that the minimum dose to the tumor margins, rather than the maximum (or prescribed) dose, may be the most important dosimetric factor for successful SRS and thereby risk of TR vs. PP. Radiation damage to the tumor induces an inflammatory response which seems to play a role in the development of PP. The more complete the target cover ratio is at SRS, the more tumor cell death is induced by radiation which again might induce a stronger antitumor immune response than if the target coverage ratio is lower.

PP is often observed in patients treated with immunotherapy.^5^ Of note, none of the patients in the cohort used to derive the Bergen Criteria were treated with immunotherapy. With their increasingly common use in a wide variety of malignancies, there may be some differences in the PP and TR percentages, depending on whether or not these drugs are being employed as systemic therapy. A retrospective study showed that immunotherapy in combination with SRS increased the local control rate and risk of radiation necrosis (RN) when immunotherapy was administered during or after SRS compared with when it was administered before SRS.^26^ Recent reviews found inconclusive differences in LC rates and risk of RN when they compared combined SRS and immunotherapy with SRS alone.^27,28^ Nevertheless, due to the potential synergistic effect of SRS in combination with checkpoint inhibitors, the predictive value of the Bergen Criteria needs to be confirmed in a cohort receiving a combination of these 2 treatments.

Tumor size is the main limitation for SRS. Smaller BM respond better to SRS and therefore also more often respond with PP than larger BM. However, BM from different primaries represent different diseases. Lung cancer BM are radiosensitive and respond well to SRS with a corresponding high rate of PP compared with BM from other primary cancer sites. Moreover, lung cancer patients have a high-risk developing BM and are thus routinely screened for BM. Consequently, BM in lung cancer patients are likely to be detected when they are smaller compared with BM in patients with more radioresistant primaries.

The significantly longer survival observed for patients experiencing TR, PP, or both compared with patients that do not suggests that the Bergen Criteria may be valid also for long-term survivors. However, as only 2 of 97 patients have more than 5 years OS in the present study, longer-term data on large datasets will need to be scrutinized to see if the Bergen Criteria are as infallible in patients who are in long-term follow-up as they appear to be in the short-term follow-up cohorts.

The strength of the study is the prospective design, the national referral area and the high compliance rate with complete MRI follow-up until death for more than 95% of the patients. Still, the relatively low number of BM included is a limitation. Secondly, the Bergen Criteria is derived in a cohort treated with SRS before the introduction of immunotherapy. This is a potential shortcoming that requires cautioning for those who might employ the Bergen Criteria in her/his own clinic to predict the future for individual patients. Thirdly, temporary volume increase on MRI-T1-C was used as definition for PP in the present study and delayed growth as definition for TR; without histological confirmation except for 2 cases with TR. Nevertheless, the volumetric curves used represent the final outcome for all BM as all patients are followed for more than 5 years or until death. Importantly, none of the BM responding with temporary volume increase (PP) recurred at a later stage. Finally, in the present study, BM are used as the observation unit and thus assessed as independent of the patient even though some patients had more than 1 BM and a dependency between BM in the same patient therefore is likely. Independent assessment of observations of BM in the same patient may lead to excessive statistical significance. Moreover, a prognostic index will always be adjusted to the material from which it has been developed with an excessively good predictability.

The Bergen Criteria is user-friendly, intuitive, and cost-free. It estimates with high accuracy the likelihood of PP, a sign of successful treatment, vs. the risk of failed treatment based on 4 simple and readily available treatment characteristics. The Bergen Criteria may be of help when deciding whether to retreat or observe, informing patient and choosing an appropriate follow-up interval. Ultimately, it may lead to reduced use of expensive PET/specialized MRI sequences and reduce the number of treatment delays and unnecessary surgeries. Larger studies are however needed to validate our results. The Bergen Criteria may be extended to additional data sources, which may lead to a refined score for subtypes of BM from different primary cancers.

## Funding

This study was supported by postdoctoral grant from Helse Vest (RHF) Regionalt samarbeidsorgan (Project number: 912042; Project title: Gamma Knife Surgery for Brain Cancer – Radiosensitizers and Imaging techniques to improve treatment Efficacy - Experimental and Clinical research.).

**Conflict of interest statement**. None declared.

## Previous presentations

Portions of this work were presented in abstract form at the SNO meeting in New Orleans in November 2018, the ISRS (International Stereotactic Radiosurgery Society) meeting in Rio in June 2019, and the SNO Inaugural Conference on Brain Metastases in New York in August 2019.

## Authorship Statement.

Experimental design: BSS, GOS; implementation of the data: BSS, GOS, PØE, P-HP, JIH; analysis and interpretation of the data: BSS, GOS, PØE, P-HP, JIH, GEE, JK.
